# Supplementary material for: SGLT2 inhibition modulates NLRP3 inflammasome activity via ketones and insulin in diabetes with cardiovascular disease
Source: Nat Commun. 2020 May 1;11:2127. doi: 10.1038/s41467-020-15983-6 (PMC7195385; doi:10.1038/s41467-020-15983-6)
Supplement: Supplementary file 1 — Supplementary Information [file 41467_2020_15983_MOESM1_ESM.pdf]

Supplementary Information

SGLT2 inhibition modulates NLRP3 inflammasome activity via ketones and insulin in diabetes with cardiovascular disease

Kim et al.

Supplementary Table 1. Adverse events for sulfonylurea and SGLT2 inhibitor groups.

Adverse events	**Sulfonylurea (*n* = 32)**	**SGLT2 inhibitor (*n* = 29)**
Any adverse event [*n* (%)]	3 (9.38)	2 (6.90)
Adverse event leading to discontinuation of a study drug [*n* (%)]	1* (3.13)	1† (3.45)
Hypoglycemia [*n* (%)]	1 (3.13)	0
Urinary tract infection [*n* (%)]	0	0
Genital infection [*n* (%)]	0	1 (3.45)
AST and/or ALT ≥2 times upper normal limit [*n* (%)]	0	0
eGFR decline ≥30% baseline [*n* (%)]	0	1‡ (3.45)
Gastrointestinal disorders [*n* (%)]	2 (6.25)	0

*Nausea; †Genital infection; ‡decline of eGFR 31% baseline. ALT, alanine aminotransferase; AST, aspartate aminotransferase; eGFR, estimated glomerular filtration rate; SGLT2, sodium-glucose cotransporter 2.

Supplementary Table 2. Effects of sulfonylurea and SGLT2 inhibitor on serum IL-1β and IL-18 levels.

	Sulfonylurea (*n* = 32)			SGLT2 inhibitor (*n* = 29)		
	**Baseline**	**Day 30**	***P***	**Baseline**	**Day 30**	***P***
Serum IL-1β (pg/mL)*	0.17 (0.08-0.53)	0.13 (0.05-0.84)	0.83	0.11 (0.07-0.35)	0.08 (0.06-0.23)	0.29
Serum IL-18 (pg/mL)	227.7 (168.8-283.0)	243.4 (181.2-266.4)	0.54	218.2 (174.4-262.8)	216.6 (164.7-286.9)	0.57

Statistical significance was evaluated by Wilcoxon signed rank test; values are described as median (interquartile range). *Not detectable (<0.033 pg/mL) in 37 (18 in sulfonylurea group and 19 in SGLT2 inhibitor group) out of 61 participants; excluded from the analyses. IL-1β, interleukin-1β; IL-18, interleukin-18; SGLT2, sodium-glucose cotransporter 2. Source data are provided as a Source Data file.

Supplementary Fig. 1. Correlation between changes in fasting serum insulin (a) or BHB (b) levels and changes in IL-1β release (*n* = 61).

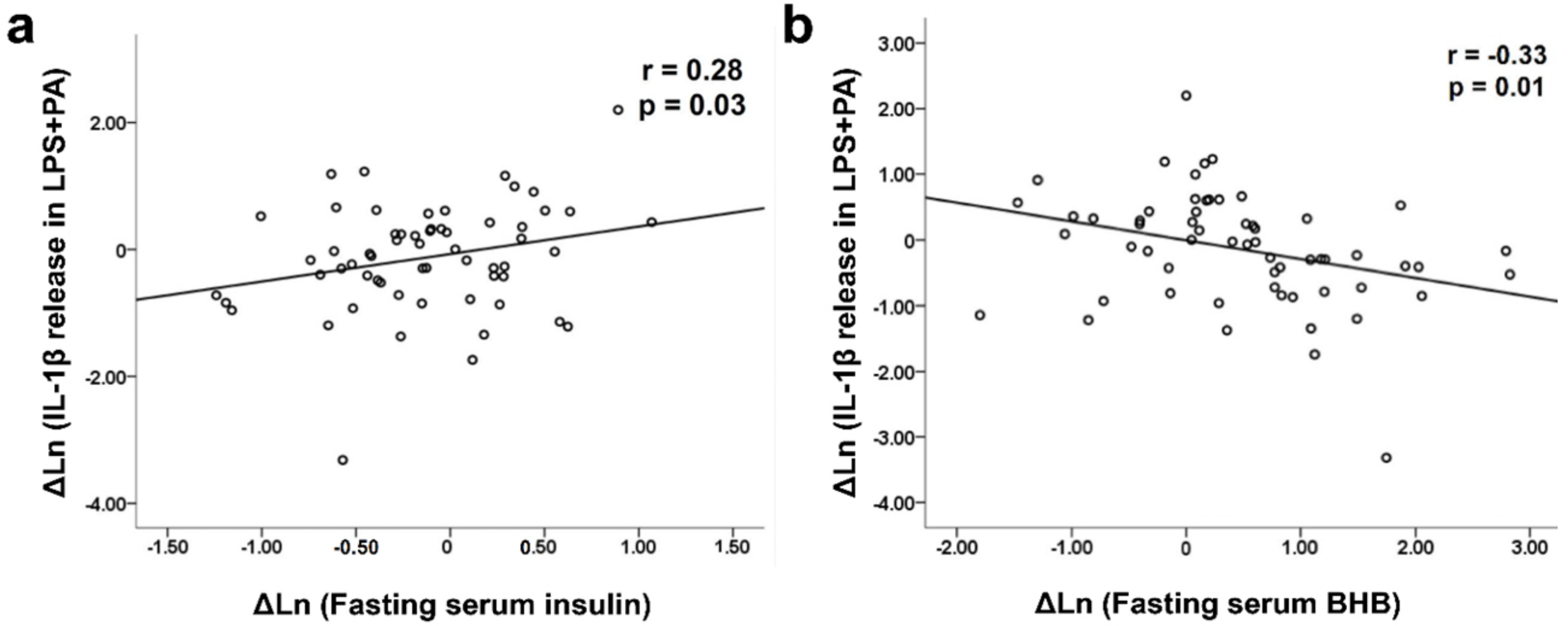

Open circles are data points from individual subjects. Pearson's correlation coefficient. Variables were log transformed for analysis. 0.1 μg/mL LPS; 0.2 mM PA. ΔLn (Fasting serum insulin) = [Ln (Fasting serum insulin at end of treatment (μU/mL)) – Ln (Fasting serum insulin at baseline (μU/mL))]; ΔLn (Fasting serum BHB) = [Ln (Fasting serum BHB at end of treatment (mM)) – Ln (Fasting serum BHB at baseline (mM))]; ΔLn (IL-1β release) = [Ln (IL-1β release at end of treatment (pg/mL)) – Ln (IL-1β release at baseline (pg/mL))]; IL-1β levels are the

mean values of measurements repeated twice or three times per sample. BHB, β-hydroxybutyrate; IL-1β, interleukin-1β; PA, palmitate.

Supplementary Fig. 2. Correlation between changes in body weight and changes in IL-1β (a) and TNF-α (b) release (*n* = 61).

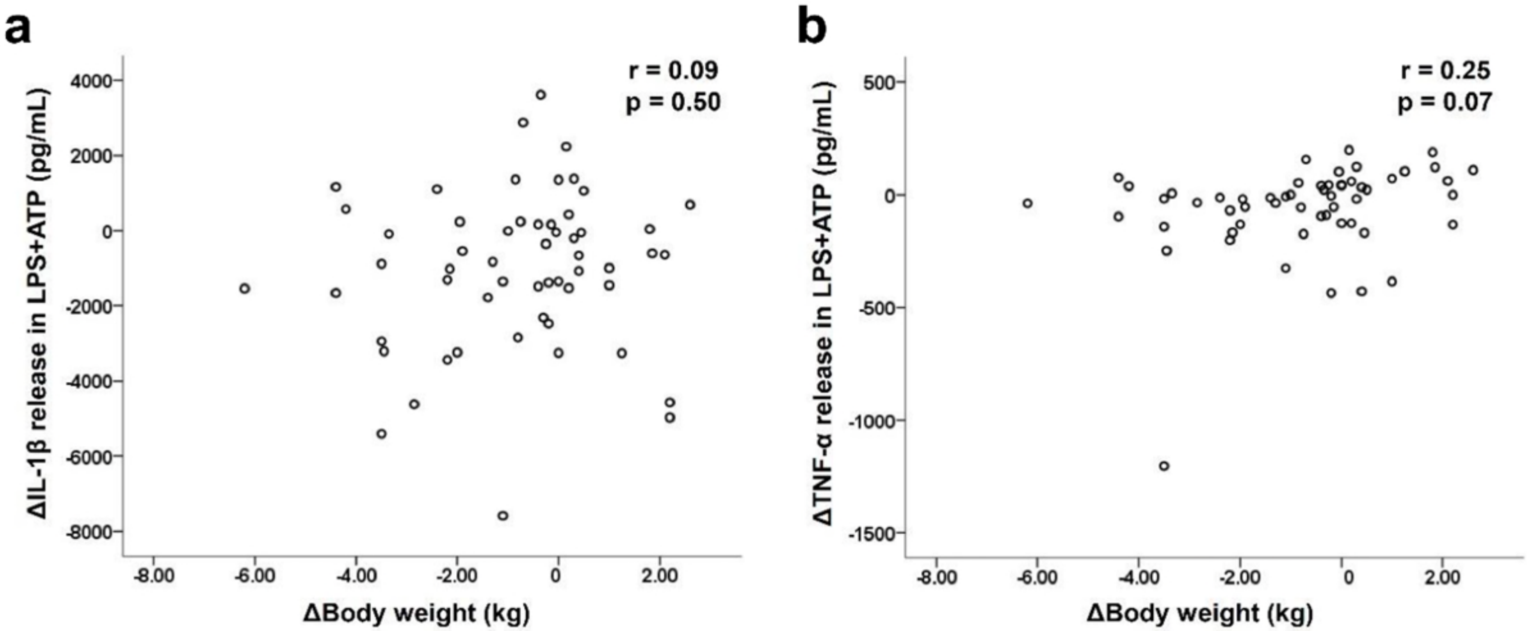

Open circles are data points from individual subjects. Pearson's correlation coefficient. 0.1 µg/mL LPS; 2 mM ATP. ΔBody weight (kg) = [Body weight at end of treatment (kg) – Body weight at baseline (kg)]; ΔIL-1β release (pg/mL) = [IL-1β release at end of treatment (pg/mL) – IL-1β release at baseline (pg/mL)]; ΔTNF-α release (pg/mL) = [TNF-α release at end of treatment (pg/mL) – TNF-α release at baseline (pg/mL)]; IL-1β and TNF-α levels are the mean values of measurements repeated twice or three times per sample. IL-1β, interleukin-1β; TNF-

α, tumor necrosis factor-α.

Supplementary Fig. 3. GO analysis for differentially expressed genes between two groups (sulfonylurea group (*n* = 3) vs. SGLT2 inhibitor group (*n* = 4)).

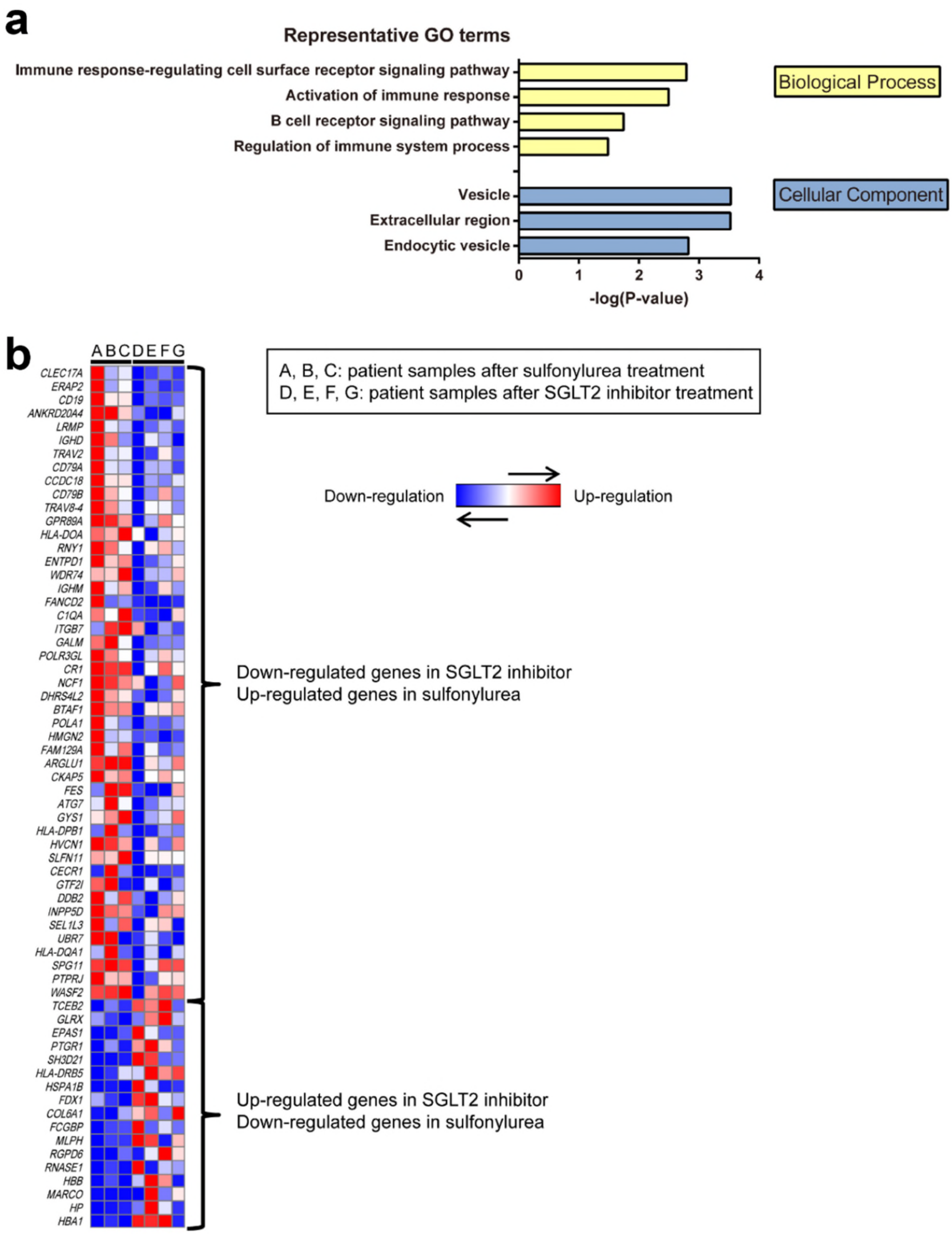

a Enrichment of biological processes in clusters of downregulated (yellow bars) and

upregulated (blue bars) macrophage genes in SGLT2 inhibitor group compared to sulfonylurea group. **b** Heatmap representation of macrophage genes of these 2 clusters. GO, gene ontology; SGLT2, sodium-glucose cotransporter 2.

Supplementary Fig. 4. Study design.

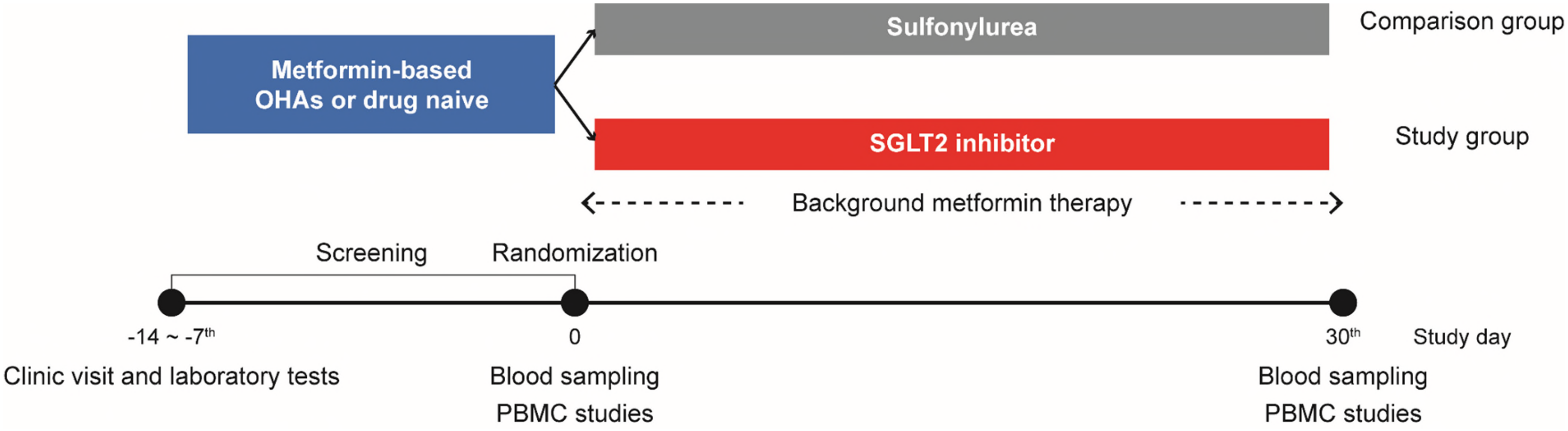

OHAs, oral hypoglycemic agents; PBMC, peripheral blood mononuclear cell; SGLT2, sodium-glucose cotransporter 2.
